# In-Vitro Evaluation of 52 Commercially-Available Essential Oils Against *Leishmania amazonensis*

**DOI:** 10.3390/molecules24071248

**Published:** 2019-03-30

**Authors:** Lianet Monzote, Isabel Herrera, Prabodh Satyal, William N. Setzer

**Affiliations:** 1 Parasitology Department, Institute of Tropical Medicine “Pedro Kouri”, Havana 10400, Cuba; monzote@ipk.sld.cu (L.M.); isa@ipk.sld.cu (I.H.); 2 Aromatic Plant Research Center, 230 N 1200 E, Suite 100, Lehi, UT 84043, USA; psatyal@aromaticplant.org; 3 Department of Chemistry, University of Alabama in Huntsville, Huntsville, AL 35899, USA

**Keywords:** leishmaniasis, frankincense, coriander, wintergreen, birch

## Abstract

Leishmaniasis is a neglected tropical disease caused by members of the *Leishmania* genus of parasitic protozoa that cause different clinical manifestations of the disease. Current treatment options for the cutaneous disease are limited due to severe side effects, poor efficacy, limited availability or accessibility, and developing resistance. Essential oils may provide low cost and readily available treatment options for leishmaniasis. In-vitro screening of a collection of 52 commercially available essential oils has been carried out against promastigotes of *Leishmania amazonensis*. In addition, cytotoxicity has been determined for the essential oils against mouse peritoneal macrophages in order to determine selectivity. Promising essential oils were further screened against intracellular *L. amazonensis* amastigotes. Three essential oils showed notable antileishmanial activities: frankincense (*Boswellia* spp.), coriander (*Coriandrum sativum* L.), and wintergreen (*Gualtheria fragrantissima* Wall.) with IC_50_ values against the amastigotes of 22.1 ± 4.2, 19.1 ± 0.7, and 22.2 ± 3.5 μg/mL and a selectivity of 2, 7, and 6, respectively. These essential oils could be explored as topical treatment options for cutaneous leishmaniasis.

## 1. Introduction

Leishmaniasis is a collection of parasitic diseases caused by several *Leishmania* species [1,2]. The disease is transmitted by several members of Phlebotominae sand flies. The genera *Lutzomyia* (New World) and *Phlebotomus* (Old World) are the only hematophagous vectors of the diseases. Leishmaniasis is considered by the World Health Organization to be a neglected tropical disease [3]. Depending on the *Leishmania* species, the disease can manifest itself in three main forms: cutaneous, mucocutaneous, and visceral. There are currently around 12–15 million people worldwide infected by *Leishmania* spp. with an estimated 350 million people at risk of acquiring leishmaniasis. Unfortunately, at the present time, there are no vaccines or prophylactic medicines available to prevent the disease. Current chemotherapeutic treatment options include sodium stibogluconate, meglumine antimoniate, conventional (deoxycholate) and liposomal amphotericin B, pentamidine isethionate, miltefosine and paromomycin. However, these agents suffer from severe side effects, poor efficacy, limited availability, high cost, or developing resistance [2,4]. Furthermore, treatment of leishmaniasis is often hampered by limited access to medicines and treatment options in developing countries where the disease is prevalent [5,6].

Essential oils are complex mixtures of volatile phytochemicals with numerous and varied biological properties [7,8]. Essential oils, particularly those that are readily available, may provide low cost treatment options for leishmaniasis. The antiparasitic activities of essential oils and their components have been demonstrated and previously reviewed [9,10]. Commercially available EOs have been screened for activity against a variety of human pathogens [11,12,13,14,15,16,17] as well as for cytotoxic activity [12,16]. In this work, we carried out in-vitro antileishmanial and cytotoxic screening of a selection of 52 commercially available essential oils against the promastigote form of *Leishmania amazonensis* and peritoneal macrophages from BALB/c, respectively. Promising antileishmanial essential oils were further screened against intracellular amastigotes.

## 2. Results and Discussion

A total of 52 commercially-available essential oils were screened for in-vitro antileishmanial activity against the promastigote form of *L. amazonensis*, as well as for cytotoxic activity against mouse peritoneal macrophages, the host cells for the amastigote form of the parasite. The antileishmanial and cytotoxic activities of the essential oils are summarized in Table 1. Of the essential oils tested, three showed notable antileishmanial activity and selectivity, frankincense oil (from the oleogum resin of *Boswellia* spp.), coriander oil (from the seeds of *Coriandrum sativum* L.), and wintergreen oil (from the leaves of *Gualtheria fragrantissima* Wall.). These three products were also active against the intracellular amastigote form of *L. amazonensis* (Table 2).

**Figure 1:**
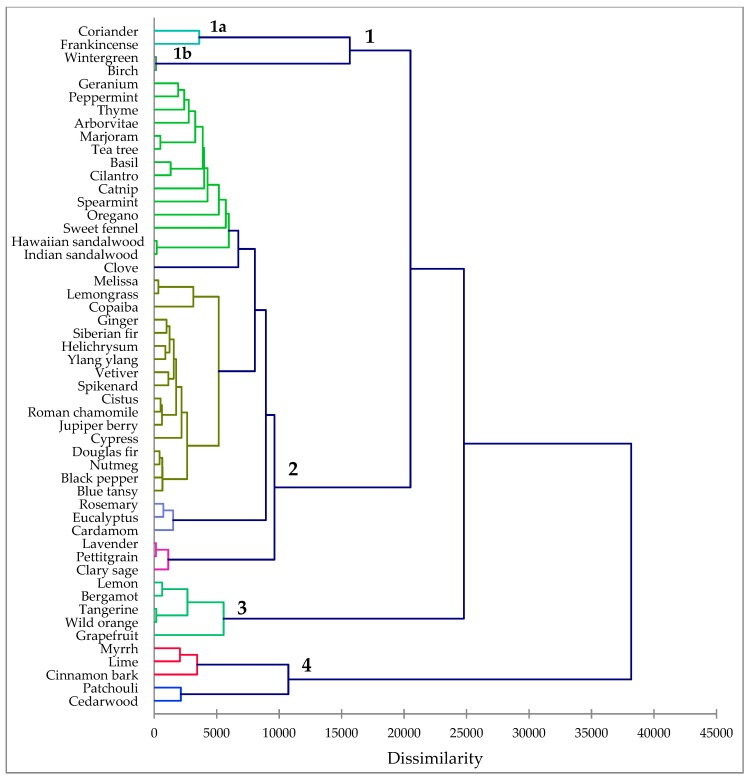
Dendrogram obtained from the agglomerative hierarchical cluster analysis of 52 commercial essential oil compositions and antileishmanial and cytotoxic activities.

The cluster analysis revealed four clusters based on biological activities (Figure 1). Cluster 1 is made up of essential oils that showed good antileishmanial activity with little or no cytotoxicity. The very large cluster 2 can be described as essential oils that showed relatively weak antileishmanial activity and relatively weak cytotoxic activity, cluster 3 is composed of essential oils with both moderate antileishmanial activity but also with moderate cytotoxic activity, and cluster 4 is made up of essential oils that were very cytotoxic.

The active cluster (Cluster 1) can be subdivided into two sub-clusters made up of coriander (*Coriandrum sativum*) and frankincense (*Boswellia* spp.), Cluster 1a, and Cluster 1b, wintergreen (*Gualtheria fragrantissima*) and birch bark (*Betula lenta* L.). Frankincense is the oleogum resin from several *Boswellia* species. Commercial frankincense sources include *B. sacra* Flueck., *B. carteri* Birdw., *B. frereana* Birdw., *B. papyrifera* Hochst. and *B. serrata* Roxb. Commercial frankincense essential oil from doTERRA International is a proprietary blend of largely *B. carteri*, followed by *B. sacra*, *B. papyrifera*, and *B. freriana*. Analysis of the essential oil used in this investigation revealed that it is composed largely of monoterpenes, including α-pinene (41.2%), α-thujene (15.7%), limonene (9.4%), sabinene (4.5%), β-pinene (3.5%), and *p*-cymene (3.3%), as well as octyl acetate (5.4%) and β-caryophyllene (3.1%). Frankincense essential oil showed activity against promastigotes and amastigotes of *L. amazonensis* with IC_50_ values of <12.5 and 22.1 ± 4.2 μg/mL, respectively. Consistent with the antiparasitic activity of frankincense essential oil, Fujisaki and co-workers screened *B. carteri* essential oil against *Plasmodium falciparum* and the oil was shown to have an IC_50_ of 10 μg/mL [18]. α-Pinene itself has shown activity against both promastigotes (IC_50_ 19.7 μg/mL) and amastigotes (IC_50_ 16.1 μg/mL) of *L. amazonensis* [19].

Another essential oil rich in α-pinene was cypress (*Cupressus sempervirens* L.) with 49.7% α-pinene. Cypress essential oil gave an IC_50_ of 40.0 μg/mL against *L. amazonensis* promastigotes, but was too cytotoxic (CC_50_ 27.2 μg/mL) against mouse peritoneal macrophages to be considered for further evaluation. Other major components in cypress essential oil were δ-3-carene (27.0%) and terpinolene (4.2%). Juniper (*Juniperus communis* L.) berry essential oil was also dominated by α-pinene (34.9%), with lesser quantities of myrcene (11.9%), sabinene (11.4%), β-pinene (7.9%), and terpinen-4-ol (4.5%). Juniper berry essential oil showed good activity against *L. amazonensis* promastigotes (IC_50_ 27.4 μg/mL) and reduced cytotoxicity against mouse peritoneal macrophages (CC_50_ 103.0 μg/mL). Thus, the presence of α-pinene seems to impart antileishmanial activity, but the activity is apparently enhanced or attenuated by other components in the essential oil.

Coriander is the fruit of *C. sativum*. Commercial coriander essential oil used in this study was obtained from dōTERRA International and the major components were linalool (73.5%), α-pinene (5.3%), and γ-terpinene (4.5%). A linalool-rich (73.2%) coriander essential oil was screened by Rondon and co-workers against *Leishmania chagasi* and was shown to be very effective against amastigotes (IC_50_ 1.51 μg/mL), but much less active against the promastigotes (IC_50_ 181 μg/mL) [20]. It is tempting to speculate that linalool might be the compound responsible for the antileishmanial activity. Indeed, Rosa and co-workers have shown linalool to be very active against both promastigotes (IC_50_ 0.0043 μg/mL) and amastigotes (IC_50_ 0.0155 μg/mL) of *L. amazonensis* [21]. However, the presence of linalool does not necessarily translate to a good antileishmanial profile. Pettitgrain (*Citrus aurantium* L.) leaf essential oil (25.4% linalool), lavender (*Lavandula angustifolia* Mill.) essential oil (34.4% linalool), and basil (*Ocimum basilicum* L.) essential oil (55.7% linalool), were only marginally active against *L. amazonensis* promastigotes (IC_50_ 56.9, 70.7, and >200 μg/mL, respectively). There may be other components in these essential oils attenuating the antileishmanial activity of linalool.

Interestingly, wintergreen (*Gualtheria fragrantissima*) essential oil, which was dominated by methyl salicylate (99.7%), showed antileishmanial activity against both *L. amazonensis* promastigotes (IC_50_ 20.7 μg/mL) and amastigotes (IC_50_ 22.2 μg/mL). *Betula lenta* (birch bark) essential oil (99.9% methyl salicylate) was somewhat less active against *L. amazonensis* promastigotes (IC_50_ 32.2 μg/mL). Considering the abundance of methyl salicylate in both wintergreen and birch essential oils, this compound is likely the active component.

Copaiba oils (not hydrodistilled essential oils) from several different species of *Copaifera* were screened by Santos and collaborators against *L. amazonensis* [22]. Antileishmanial activities against the promastigotes ranged from 5.0 to 22.0 μg/mL. Consistent with these results, copaiba oil in this study (50% β-caryophyllene) showed an IC_50_ of 17.2 μg/mL. The copaiba essential oil, however, was also too cytotoxic (CC_50_ 23.3 μg/mL) to warrant further consideration. On the other hand, β-caryophyllene was found to be remarkably active against *L. amazonensis* amastigotes (IC_50_ 1.3 μg/mL) but less toxic to murine macrophages (CC_50_ 63.6 μg/mL) [23]. In contrast, antipromastigote activity of β-caryophyllene on *L. amazonensis* was reported to have an IC_50_ of 19 μg/mL [24]. β-Caryophyllene was also found to show excellent antileishmanial activity against *L. infantum* and *L. major* (IC_50_ 1.06 and 1.33 μg/mL, respectively), with less cytotoxicity against Raw 264.7 mouse macrophage cells [25].

Rosemary (*Rosmarinus officinalis* L.) essential oil from Colombia (composition not reported) showed activity against *L. braziliensis* promastigotes with an IC_50_ of 17.4 μg/mL [26]. Similarly, rosemary essential oil from Tunisia (43.8% 1,8-cineole, 12.0% camphor, 11.5% α-pinene, 8.6% β-pinene, 4.8% camphene) was active against *L. infantum* (IC_50_ 16.3 μg/mL) and *L. major* (IC_50_ 20.9 μg/mL) promastigotes [25]. Conversely, a commercial rosemary essential oil obtained in Germany was inactive against *L. major* promastigotes (IC_50_ 282 μg/mL) [11]. In this present study, commercial rosemary essential oil (45.9% 1,8-cineole, 12.0% α-pinene, 10.9% camphor, 6.3% β-pinene) was only marginally antileishmanial (IC_50_ 89.7 μg/mL), but also marginally cytotoxic (CC_50_ 83.4 μg/mL). Rosemary essential oils have shown great variation in composition with at least five different chemotypes, which likely result in different biological activities [27].

Mikus and co-workers have screened several essential oils and essential oil components for antileishmanial activity against *L. major* promastigotes [11]. These workers found commercial *Melissa officinalis* L. essential oil to show excellent activity against *L. major* promastigotes (IC_50_ 7.0 μg/mL) with less toxicity against HL-60 (human leukemia) cells (CC_50_ 25.5 μg/mL). Andrade and co-workers screened a commercial *M. officinalis* essential oil (37.2% neral, 52.0% geranial) and fount marginal activity against *L. amazonensis* promastigotes with an IC_50_ of 132 μg/mL [17]. In contrast, *M. officinalis* essential oil from Colombia was inactive against *L. braziliensis* promastigotes [26]. In this present work, *M. officinalis* essential oil showed antileishmanial activity (IC_50_ 24.6 μg/mL) but also cytotoxicity (CC_50_ 37.3 μg/mL) giving it an unfavorable selectivity index. The major components in *M. officinalis* essential oil were β-caryophyllene (13.4%), geranial (30.2%), and neral (23.1%). Citral, a mixture of geranial and neral, has shown antileishmanial activity against *L. infantum*, *L. tropica*, and *L. major* promastigotes with IC_50_ values of 42, 34, and 36 μg/mL, respectively [28]. However, citral has demonstrated in-vitro cytotoxic activity against several cell lines [29,30,31]. There are several chemotypes of *M. officinalis* based on essential oil composition, which likely account for the differences in biological activities. Nevertheless, the citral chemotype of *M. officinalis* has also shown in-vitro cytotoxicity [32].

Likewise, the unfavorable bioactivity profile of lemongrass (*Cymbopogon flexuosus* (Nees) Will. Watson) essential oil in this study is likely due to the abundance of geranial (49.9%) and neral (23.4%). However, Machado and co-workers found that citral-rich *C. citratus* as well as citral did not exhibit cytotoxic effects on either primary bovine aortic endothelial cells or RAW 264.7 macrophage cells [28]. Similarly, Santin and co-workers found both *C. citratus* essential oil and citral to be antileishmanial against promastigotes (IC_50_ 1.7 and 8.0 μg/mL, respectively) and amastigotes (IC_50_ 3.2 and 25.0 μg/mL, respectively) of *L. amazonensis*, but with lower cytotoxicity against J774G8 macrophage cells [33].

As has been appreciated, many pure components present in the tested EOs have exhibited some level of antileishmanial activity. In fact, it is known that the compounds present in the EOs can act synergistically in mixtures, increasing the intrinsic effects of the purified components. Therefore, we suggest the potential use of EOs as mixtures. In addition, we have also taken into account that the tested EOs are commercially available in their present forms, reducing the cost of a therapeutic alternative, which constitutes one of main drawbacks of current antileishmanial treatments. The notable antileishmanial effects and moderate cytotoxicities in the case of mixtures of compounds could be further explored in animal models of cutaneous leishmaniasis by intralesional or topical routes.

## 3. Materials and Methods 

### 3.1. Essential Oils

The essential oils were obtained from commercial sources, dōTERRA International (Pleasant Grove, UT, USA), Ameo Essential Oils (Zija International, Lehi, UT, USA), Mountain Rose Herbs (Eugene, OR, USA), or Albert Vielle (Vallauris, France) and were previously analyzed by gas chromatography–mass spectrometry.

### 3.2. Parasites

*Leishmania amazonensis* parasites (Reference strain MHOM/77/BR/LTB0016) were kindly provided by the Department of Immunology, Oswaldo Cruz Foundation (FIOCRUZ), Brazil. The parasites were routinely isolated from mouse lesions (BALB/c mice) and maintained as promastigotes at 26 °C in Schneider’s medium (Sigma-Aldrich, St. Louis, MO, USA) containing 10% heat-inactivated fetal bovine serum (HFBS) (Sigma-Aldrich, St. Louis, MO, USA) and 100 U of penicillin and 100 μg streptomycin per mL as antibiotics. Parasite cultures were passaged every 3–4 days, but no longer used after the tenth passage after isolation from mice.

### 3.3. In-vitro Anti-promastigote Screening

Screening against *L. amazonensis* promastigotes was carried out as previously described [34,35,36]. Into each well in a 96-well plate, 50 μL medium (Schneider’s medium + HFBS + antibiotics) was added. Into the wells of column 2 and 7, 48 μL medium + 2 μL sample (dimethylsulfoxide solutions of essential oil, 20 mg/mL) were added. Five two-fold serial dilutions were carried out down each column to give final test concentrations of 12.5, 25, 50, 100 and 200 μg/mL. Exponentially growing parasites (4 × 10^5^) in medium were added to each well. Dimethylsulfoxide (DMSO) served as the negative control and pentamidine (Richet, Buenos Aires, Argentina) was used as a positive control. The plates were sealed with Parafilm^®^ and incubated at 26 °C for 72 h. Afterward, 20 μL of a solution (5 mg/mL) of MTT (3-[4,5-dimethylthiazol-2-yl]-2,5-diphenyltetrazolium bromide) (Sigma-Aldrich, St. Louis, MO, USA) was added to each well. The plates were incubated for an additional 4 h, the supernatant was removed, and the formazan crystals were dissolved with 100 μL DMSO. The absorbance of each well was determined with a plate reader (Molecular Devices, San Jose, CA, USA) with a test wavelength of 560 nm and a reference wavelength of 630 nm from which median inhibitory concentrations (IC_50_) were calculated [37,38]. The IC_50_ values were determined from linear dose-response curves fit to the data using a linear equation model. Each test was carried out in triplicate, and the results expressed as means ± standard deviations (SD). 

### 3.4. Mouse Peritoneal Macrophage Cytotoxicity Screening

The median cytotoxic concentrations (CC_50_) of the essential oils on mouse peritoneal macrophages (the host cells of *Leishmania* parasites) were determined as previously described [34,35,36]. Macrophages were collected from the peritoneal cavities of normal BALB/c mice and suspended in ice-cold RPMI 1640 medium (Sigma-Aldrich, St. Louis, MO, USA), supplemented with antibiotics. The cells were seeded in 96-well plates (3 × 10^5^ cells/well) and incubated at 37 °C for 2 h. Non-adherent cells were removed. Into the wells of column 2 and 7, 2 μL of essential oil solutions (as above) and 48 μL medium (supplemented with 10% HFBS and antibiotics) were also added. Two-fold serial dilutions down each lane were carried out to give final concentrations of 12.5–200 μg/mL. The macrophages were incubated for 72 h, after which the cytotoxicity was determined using the MTT assay (as above), using 15 μL/well. The CC_50_ was calculated for each compound in the same manner as the IC_50_ and selectivity indices (SI) for each essential oil were determined (CC_50_ for macrophages / IC_50_ for promastigotes). 

Essential oils with an IC_50_ < 100 µg/mL and a SI ≥ 5 were selected for further evaluation against *L. amazonensis* amastigotes [34].

### 3.5. In-vitro Intracellular Anti-amastigote Screening

Median inhibitory concentrations (IC_50_) of the active essential oils on *L. amazonensis* amastigotes were carried out as previously described [34,35,36]. The peritoneal macrophages, obtained from BALB/c mice (as above), were seeded in 24-well culture plates at 10^6^ cells/mL. The plates were incubated at 37 °C under a 5% CO_2_ atmosphere for 2 h. Non-adherent cells were removed, and stationary-phase *L. amazonensis* promastigotes were added (4:1 promastigote/macrophage ratio), and incubation continued for an additional 4 h. Free parasites were removed and 1000 μL RPMI completed medium was added to each well. Into the first well, 990 μL medium and 10 μL essential oil solution were added. Four two-fold dilutions were carried out to give final concentrations of 12.5, 25, 50 and 100 μg/mL. The plate was incubated at 37 °C under a 5% CO_2_ atmosphere for 48 h. The cell monolayers were fixed in absolute methanol, stained with Giemsa, and evaluated using a light microscope. The number of intracellular amastigotes was determined by counting 25 macrophages per sample. The results are expressed as percent reduction of infection rate (% infected macrophages × number amastigotes per infected macrophage) compared to that of the controls. The IC_50_ values were determined from the concentration-response linear curves. Each evaluation was carried out in triplicate and the results expressed as means ± SD.

### 3.6. Hierarchical Cluster Analysis

The chemical compositions of the commercial essential oils along with the antileishmanial and cytotoxic activities were used in an agglomerative hierarchical cluster (AHC) analysis. The essential oil compositions of the 52 commercially-available essential oils and the bioactivities were used to determine the associations between the essential oils and their activities using XLSTAT Premium, version 2018.5.53172 (Addinsoft, Paris, France). Dissimilarity was determined using Euclidean distance, and clustering was defined using Ward’s method.

## 4. Conclusions

This study has shown that the essential oils of frankincense, coriander, wintergreen, and birch have notable antiparasitic activities against *Leishmania amazonensis* with an acceptable SI with respect to cytotoxicity against mouse peritoneal macrophages. However, essential oils are complex mixtures of compounds, and the biological activities cannot necessarily be attributed to individual components; there are apparent synergistic and/or antagonistic interactions as well. Nevertheless, essential oils containing appreciable concentrations of α-pinene, linalool, or methyl salicylate should be investigated for antiparasitic activity.

## Figures and Tables

**Table 1:** Screening of 52 essential oils against *Leishmania amazonensis* promastigotes and BALB/c mouse peritoneal macrophages.

Essential Oil	Commercial Source	IC_50_ ± SD (μg/mL) Promastigotes *L. amazonensis*	CC_50_ ± SD (μg/mL) Macrophage from BALB/c mice	Selectivity Index (SI)	Comments
*Abies sibirica* Ledeb. (Siberian fir)	dōTERRA	58.2 ± 8.5	42.5 ± 2.4	1	Unspecific
*Anthemis nobilis* L. (Roman chamomile)	dōTERRA	27.9 ± 4.5	85.7 ± 6.0	3	Unspecific
*Betula lenta* L. (birch)	dōTERRA	32.2 ± 6.6	136.8 ± 9.2	4	Unspecific
***Boswellia*** **Roxb. ex Colebr. spp. (frankincense)**	**dōTERRA**	**<12.5**	**59.3 ± 1.8**	**5**	**Active/Follow up**
*Cananga odorata* (Lam.) Hook. f. & Thomson (ylang ylang)	dōTERRA	36.6 ± 3.9	55.6 ± 0.5	2	Unspecific
*Cinnamomum zeylanicum* Blume (cinnamon bark)	dōTERRA	<12.5	<12.5	-	Too Toxic
*Cistus ladanifer* L. (cistus)	Albert Vielle	19.2 ± 4.6	56.9 ± 8.1	3	Unspecific
*Citrus aurantium* L. (pettitgrain)	dōTERRA	56.9 ± 1.8	77.4 ± 0.9	1	Unspecific
*Citrus aurantium* L. (wild orange)	dōTERRA	34.8 ± 2.2	35.2 ± 1.7	1	Unspecific
*Citrus aurantifolia* Swingle (lime)	dōTERRA	<12.5	<12.5	-	Too toxic
*Citrus* × *bergamia* Risso & Poit. (bergamot)	dōTERRA	59.2 ± 6.3	44.9 ± 1.6	1	Unspecific
*Citrus limon* (L.) Osbeck (lemon)	dōTERRA	71.2 ± 1.9	71.1 ± 1.6	1	Unspecific
*Citrus* × *paradisi* Macfad. (grapefruit)	dōTERRA	35.5 ± 4.4	<12.5	-	Too Toxic
*Citrus reticulata* Blanco (tangerine)	dōTERRA	70.7 ± 7.8	54.3 ± 1.0	1	Unspecific
*Commiphora myrrha* (T. Nees) Engl. (myrrh)	dōTERRA	<12.5	<12.5	-	Too Toxic
***Coriandrum sativum* L. (coriander)**	**dōTERRA**	**<12.5**	**141.7 ± 4.1**	**>8**	**Active/Follow up**
*Coriandrum sativum* L. (cilantro)	dōTERRA	34.4 ± 4.8	45.9 ± 1.3	1	Unspecific
*Copaifera* L. spp. (copaiba)	dōTERRA	17.2 ± 0.1	23.3 ± 7.1	1	Unspecific
*Cupressus sempervirens* L. (cypress)	Améo	40.0 ± 2.3	27.2 ± 0.7	1	Unspecific
*Cymbopogon flexuosus* (Nees) Will. Watson (lemongrass)	dōTERRA	27.8 ± 0.4	30.0 ± 2.0	1	Unspecific
*Elettaria cardamomum* (L.) Maton (cardamom)	dōTERRA	109.5 ± 1.3	60.5 ± 6.0	1	Unspecific
*Eugenia caryophyllata* Thunb. (syn. *Syzygium aromaticum* (L.) Merr. & L.M. Perry) (clove)	dōTERRA	>200	143.1 ± 11.9	-	Inactive
*Eucalyptus radiata* Sieber ex DC. (eucalyptus)	dōTERRA	164.7 ± 8.3	100.2 ± 8.4	1	Unspecific
*Foeniculum vulgare* Mill. (sweet fennel)	dōTERRA	>200	66.9 ± 7.9	-	Inactive
***Gualtheria fragrantissima* Wall.(wintergreen)**	**dōTERRA**	**20.7 ± 1.6**	**135.4 ± 5.7**	**7**	**Active/Follow up**
*Helichrysum italicum* G. Don f. (helichrysum)	Améo	42.8 ± 2.2	55.2 ± 2.4	1	Unspecific
*Juniperus communis* L. (juniper berry)	Améo	27.4 ± 0.5	103.0 ± 9.3	4	Unspecific
*Juniperus virginiana* L. (cedarwood)	dōTERRA	53.2 ± 8.4	<12.5	-	Too Toxic
*Lavandula angustifolia* Mill. (lavender)	Améo	70.7 ± 5.0	82.3 ± 2.4	1	Unspecific
*Melaleuca alternifolia* Cheel (tea tree)	dōTERRA	70.7 ± 6.2	42.5 ± 1.4	1	Unspecific
*Melissa officinalis* L. (melissa)	dōTERRA	24.6 ± 0.7	37.3 ± 1.4	2	Unspecific
*Mentha piperita* L. (peppermint)	dōTERRA	172.8 ± 4.1	67.3 ± 4.4	0	Unspecific
*Mentha spicata* L. (spearmint)	dōTERRA	79.8 ± 3.0	90.4 ± 5.6	1	Unspecific
*Myristica fragrans* Houtt. (nutmeg)	Améo	133.5 ± 2.6	40.0 ± 1.1	0	Unspecific
*Nardostachys jatamansi* (D. Don) DC. (spikenard)	dōTERRA	49.8 ± 1.4	22.1 ± 4.0	0	Unspecific
*Nepeta cataria* L. (catnip)	Mountain Rose	54.0 ± 4.4	81.6 ± 0.8	2	Unspecific
*Ocimum basilicum* L. (basil)	dōTERRA	>200	69.1 ± 9.0	-	Inactive
*Origanum majorana* L. (marjoram)	dōTERRA	>200	25.7 ± 1.3	-	Inactive
*Origanum vulgare* L. (oregano)	dōTERRA	>200	66.5 ± 0.9	-	Inactive
*Pelargonium graveolens* L'Hér. ex Aiton (geranium)	Améo	>200	57.4 ± 3.6	-	Inactive
*Piper nigrum* L. (black pepper)	dōTERRA	57.7 ± 3.7	35.6 ± 5.7	1	Unspecific
*Pogostemon cablin* (Blanco) Benth. (patchouli)	Améo	68.7 ± 7.8	<12.5	-	Too Toxic
*Pseudotsuga menziesii* (Mirb.) Franco (Douglas fir)	dōTERRA	82.5 ± 4.5	37.7 ± 3.2	0	Unspecific
*Rosmarinus officinalis* L. (rosemary)	dōTERRA	89.7 ± 2.0	83.4 ± 7.3	1	Unspecific
*Santalum album* L. (Indian sandalwood)	dōTERRA	105.5 ± 6.0	29.9 ± 6.3	0	Unspecific
*Santalum paniculatum* Hook. & Arn. (Hawaiian sandalwood)	dōTERRA	43.1 ± 2.2	25.9 ± 5.3	1	Unspecific
*Salvia sclarea* L. (clary sage)	Améo	>200	58.6 ± 9.0	-	Inactive
*Tanacetum annuum* L. (blue tansy)	dōTERRA	52.2 ± 2.8	36.6 ± 5.8	1	Unspecific
*Thuja plicata* Donn ex D. Don (arborvitae)	dōTERRA	67.1 ± 3.1	61.9 ± 6.1	1	Unspecific
*Thymus vulgaris* L. (thyme)	dōTERRA	>200	30.5 ± 5.5	-	Inactive
*Vetiveria zizanioides* (L.) Nash (syn. *Chrysopogon zizanioides* (L.) Roberty) (vetiver)	dōTERRA	19.0 ± 3.3	31.7 ± 2.8	2	Unspecific
*Zingiber officinale* Roscoe (ginger)	dōTERRA	39.9 ± 3.4	58.3 ± 4.7	1	Unspecific
Pentamidine		0.37 ± 0.01	11.7 ± 1.7	31	**Active**

IC_50_: Median inhibitory concentration. Concentration that inhibits 50% of promastigote growth. CC_50_: Median cytotoxic concentration. Concentration that causes the mortality of 50% of macrophages. SD: Standard deviation. SI: Selectivity index: CC_50_/IC_50._ In bold, essential oils that were selected as active and selective.

**Table 2:** Screening of frankincense, coriander, and wintergreen essential oils against *Leishmania amazonensis* intracellular amastigotes.

Essential Oil	IC_50_ ± SD (µg/mL)	SI
*Boswellia* spp. (frankincense)	22.1 ± 4.2	2
*Coriandrum sativum* (coriander)	19.1 ± 0.7	7
*Gualtheria fragrantissima* (wintergreen)	22.2 ± 3.5	6
Pentamidine	1.3 ± 0.1	9

IC_50_: Median inhibitory concentration. Concentration that inhibits 50% of promastigote growth. SD: Standard deviation. SI: Selectivity index: CC_50_/IC_50._
